# Unveiling the hidden link between oral flora and colorectal cancer: a bidirectional Mendelian randomization analysis and meta-analysis

**DOI:** 10.3389/fmicb.2024.1451160

**Published:** 2024-09-10

**Authors:** Zexin Zhang, Wenfeng Wu, Zhikai Xiahou, Yafeng Song

**Affiliations:** ^1^The Second Clinical School of Guangzhou University of Chinese Medicine, Guangzhou, China; ^2^China Institute of Sport and Health Science, Beijing Sport University, Beijing, China

**Keywords:** causal relationship, oral flora, colorectal cancer, saliva, tongue, Mendelian analysis, meta-analysis

## Abstract

**Objective:**

The impact of oral flora on intestinal micro-environment and related diseases has been widely reported, but its role in colorectal cancer (CRC) remains elusive.

**Methods:**

A Two-sample Mendelian Randomization (TSMR) analysis was conducted to explore the causal relationship between oral flora and CRC, with the Inverse-Variance Weighted (IVW) serving as the primary method for evaluating this causal relationship. Data on the oral flora were derived from human samples from the tongue and saliva, with all cohort populations originating from Asia. In addition, 2 independent external cohorts were used to validate the positive results and perform a meta-analysis of the final results. Lastly, to balance the effect of positive oral flora on CRC, a Multivariate Mendelian Randomization (MVMR) analysis was also performed.

**Results:**

The TSMR analysis revealed that 17 oral flora may have a causal relationship with CRC in the training cohort. Among them, *s Haemophilus, g Fusobacterium, s Metamycoplasma salivarium,* and *s Mogibacterium pumilum* were validated in two testing cohorts. Intriguingly, after integrating the results of the 3 cohorts for meta-analysis, 16 associations remained significant. In the training cohort, MVMR analysis demonstrated that *s Capnocytophaga ochracea* and *s Metamycoplasma salivarium* retained statistical significance. In one of the testing cohorts, *s Metamycoplasma salivarium, s Streptococcus anginosus,* and *s Streptococcus sanguinis* retained statistical significance. In the other testing cohort, *s Metamycoplasma salivarium, s Haemophilus,* and *g Fusobacterium* remained significant.

**Conclusion:**

*s Haemophilus, g Fusobacterium, s Metamycoplasma salivarium, and s Mogibacterium pumilum* have a solid causal relationship with the occurrence and development of CRC.

## Background

As is well documented, colorectal cancer (CRC) is the third leading cause of cancer-related mortality worldwide (Sung et al., 2021). Among gastrointestinal tumors, CRC-related mortality can be as high as 48.1% (Jinjuvadia et al., 2013). Given the subtle nature of early symptoms and challenges in detection, the majority of CRC patients are diagnosed at advanced stages (Johnston, 2005). At present, surgery, chemotherapy, and radiotherapy remain the primary treatments for CRC (Sun et al., 2018). While the survival rate of CRC patients has been significantly improved with the advent of targeted and immunotherapy (Fridman et al., 2020), their survival time remains suboptimal. Moreover, the etiology of CRC remains to be elucidated. Although changes in dietary habits, obesity, and aging have been identified as high-risk factors, CRC is typically not driven by a single mechanism (Cueva et al., 2020). Therefore, there is a pressing need to identify effective strategies for the treatment and prevention of CRC.

Oral flora is one of the most complex microenvironments in the human body, containing various microorganisms such as various bacteria and fungi (He and Shi, 2009). Primarily distributed in the tongue and saliva (Hull and Chow, 2007), these microorganisms can colonize the intestines of susceptible individuals (Atarashi et al., 2017; Schmidt et al., 2019; Liu et al., 2021a). Notably, a healthy individual swallows 1–1.5 L of saliva every day, which eventually reaches the human gastrointestinal tract (Humphrey and Williamson, 2001). Therefore, oral flora is closely related to human health, especially for digestive tract diseases (Guha et al., 2007). Numerous studies have established that the oral cavity acts as an extragastric reservoir for *Helicobacter pylori* (*H. pylori*) (Wei et al., 2019; Avcu et al., 2001; Dye et al., 2002). This may account for the infectious process of *H. pylori*, which initially colonizes the oral cavity and migrates to the stomach (Yee, 2017). Gastric *H. pylori* infection has been identified as a high-risk factor for gastric cancer (Yee, 2017). Given that the colorectum is the terminal segment of the digestive system, exploring changes in oral flora is crucial, considering that it may play a pivotal role in intestinal diseases.

In recent years, an increasing number of studies have documented an association between oral flora and the occurrence and progression of CRC. The predominant phyla of oral bacteria comprise *Firmicutes, Bacteroidetes, Proteobacteria, Actinobacteria*, and *Fusobacteria*. In several studies, *Fusobacterium nucleatum* and *Porphyromonas* spp. have been reported to promote the occurrence and development of CRC (Cho et al., 2014; Vipperla and O’Keefe, 2016; Yang et al., 2017). In addition, an increase in the abundance of clostridium species is typically regarded as a risk factor for poor prognosis in CRC (Kostic et al., 2012). Indeed, a decrease in intestinal microbiota and a concomitant increase in clostridium species is a common characteristic in CRC patients (Koliarakis et al., 2019). The close relationship between the oral flora and CRC may be ascribed to the metabolic substances synthesized by the former. For instance, Paps2, FadA, and LPS secreted by clostridium species possess oncogenic properties (Cueva et al., 2020). Despite ongoing research on the association between oral flora and CRC, studies examining this link remain scarce. In addition, the number of oral flora is large and extends beyond saliva. The tongue also harbors a substantial number of underexplored microorganisms. On the other hand, although there are reports investigating the link between oral flora and CRC, their conclusions are contradictory, warranting further in-depth exploration.

Mendelian Randomization (MR) analysis is a method for evaluating causal associations and is widely applied in research fields such as epidemiology (Birney, 2022). Given that alleles of parental genetic variants are randomly assigned, MR analysis can simulate the randomization process in randomized controlled trials, thereby mitigating the influence of confounding factors and reverse causality in observational studies and enhancing the reliability of causal inferences between exposures and outcomes (Sekula et al., 2016).

In this study, a Two-sample Mendelian Randomization (TSMR) analysis was performed to identify oral flora that is causally associated with CRC. Next, two external cohorts were utilized to validate the positive results. In order to enhance the reliability of the results, a meta-analysis was performed by integrating the results of the three cohorts. Then, a Multivariate Mendelian Randomization (MVMR) analysis was conducted to account for the effects of different oral flora on CRC. Finally, the robustness of the results was ensured through Reverse Mendelian Randomization (RMR) analysis.

## Methods and materials

### Data acquisition and process

The SNP data of oral flora and CRC were obtained from Asian populations. The GWAS summary data of oral flora was derived from a study published by Liu et al. (2021b). This large-scale metagenomic-genome-wide association study (mgGWAS) analyzed 2017 tongue dorsum samples and 1915 saliva samples from 2,984 healthy individuals and provided high-depth whole-genome sequencing data. The present study included a total of 1,549 saliva samples and 1,568 tongue samples. Additionally, the associations were validated in an independently replicated cohort consisting of 1,494 individuals.

The SNP data of CRC in the training cohort were sourced from BioBank Japan, with all samples also originating from Asian populations (Sakaue et al., 2021). In this study, 167,691 samples were included, comprising 159,386 control samples and 8,305 disease samples. The samples included both male and female individuals. Finally, a total of 12,456,388 SNPs were obtained from these samples.

### Screening of instrumental variables

Initially, to identify instrumental variables (IVs) with a strong correlation to oral flora, a filtering threshold of P1 = 5e-5 was applied for single nucleotide polymorphisms (SNPs), which are typically detected by whole-genome sequencing analysis, with each SNP having a corresponding *p* value. In the MR analysis, SNPs that are highly correlated with the exposure assist in ensuring robust results. SNPs exhibiting greater significance levels are deemed to be closely associated with the heritability of oral flora. Furthermore, an F test was conducted on each IV to exclude weak IVs. The F-test, based on improving’ first-order’ weights and’ second-order’ weights, assists in preventing the inflation and undetectability of heterogeneity. The F test formula is expressed as F = (Beta/Se)^2^, where Beta represents the effect size of IVs on oral flora and Se denotes the standard error associated with Beta. IVs with a value below 10 in the F test were excluded from the study. Additionally, a linkage disequilibrium assessment was conducted on the instrumental variables. Linkage disequilibrium in genetics reflects the likelihood of alleles from multiple gene loci co-occurring on a single chromosome at a frequency greater than that expected by chance. Such occurrences can introduce bias in Mendelian randomization analysis. To mitigate this bias, a threshold of *r*^2^ = 0.001 and Kb = 10,000 was applied.

The IVs of CRC were extracted concurrently with the IVs of the oral flora following a screening process. A threshold of P2 = 5e-5 was set for the IVs of the outcome in order to eliminate highly correlated variables from the outcome data. No proxy tools were employed for IVs absent in the CRC dataset to ensure the accuracy and reliability of the results. Subsequently, data for IVs associated with the oral flora and CRC were combined, while palindromic SNPs were excluded.

### Two sample MR analysis

The TwosampleMR package in R was utilized for conducting the comprehensive MR analysis on the merged data. The MR analysis encompassed four distinct methods, including the Inverse Variance Weighted (IVW), MR Egger, Weighted Median, and Weighted Mode. The results obtained from the IVW method were primarily relied upon for evaluation owing to its ability to detect bias even in the presence of invalid IVs (Burgess et al., 2015). In contrast, the MR Egger method introduces an intercept to assess and address horizontal pleiotropy in IVs (Bowden et al., 2015). To ensure the robustness of the results, only results with the same Beta direction across the four analysis methods were retained, whereas unclassified oral flora was excluded in this study.

### Multivariate Mendelian randomization analysis

To elucidate the involvement of various oral flora in CRC and boost the validity of the TSMR findings, Multivariate Mendelian Randomization (MVMR) analysis was employed to assess the potential link between oral flora and CRC (Burgess and Thompson, 2015). Initially, single nucleotide polymorphisms (SNPs) shared among several oral flora types were identified and subsequently isolated from the dataset. Following the elimination of linkage disequilibrium, the extracted SNP data were subjected to MVMR analysis. The IVW method, similar to TSMR, can evaluate the principal outcome of MVMR. Additionally, Supplementary materials such as MR Egger, Lasso, and Weighted median were employed to enhance the robustness of the IVW results. Heterogeneity testing was conducted utilizing the IVW method, while pleiotropic effects were assessed by examining the Egger intercept and MR Presso.

### Verification based on testing cohort and meta-analysis

To mitigate the influence of individual study outcomes on MR results, a meta-analysis was conducted on data from other two distinct cohorts of CRC patients to determine the effect size of oral flora on CRC. The meta-analysis primarily relied on the IVW method to assess the association between oral flora on CRC analyses. In the meta-analysis, heterogeneity was evaluated using the *I*^2^ statistics. An *I*^2^ value less than or equal to 50% indicates low to medium heterogeneity, and the fixed-effect model is adopted. In contrast, *I*^2^ values exceeding 50% indicated high heterogeneity, and the random-effects model was used. The formula for calculating *I*^2^ is as follows: *I*^2^ = 100% × (Q-df)/Q, where Q represents Cochran’s Q heterogeneity statistic, and df denotes the degree of freedom.

### Statistics analysis

Horizontal pleiotropy significantly compromises the validity of MR analysis findings by allowing IVs to affect outcomes through multiple genetic pathways, thereby contravening the fundamental principles of MR analysis. IVs with horizontal pleiotropy usually signify that SNPs have multiple genetic functions, which can affect the reliability and accuracy of conclusions. To identify and address horizontal pleiotropy, the MR-Presso (Verbanck et al., 2018) and MR Egger methods were employed to assess IVs for potential pleiotropic effects. IVs that passed both tests were deemed free from horizontal pleiotropy, whereas those exhibiting pleiotropic effects were excluded from the analysis. Furthermore, heterogeneity among IVs was examined using the IVW and MR Egger methods. IVs with significant heterogeneity were subsequently excluded from the analysis.

A leave-one-out sensitivity analysis was conducted for each IV to assess the potential influence of individual SNPs on the outcome. Sequentially eliminating SNPs and observing the MR effect size of the remaining SNPs on the outcome can assist in identifying single SNPs that significantly influence the outcome, which is conducive to ensuring that each SNP contributes uniformly to the MR analysis. The Steiger test was utilized to identify and exclude SNPs exhibiting reverse causality, given that these can distort the interpretation of the relationship between exposure and outcome. Additionally, a Reverse Mendelian randomization (RMR) analysis was performed between CRC and oral flora to further validate the findings.

## Results

### Characteristics of SNPs

A total of three CRC datasets were retrieved from the GWAS summary data, with IDs as follows: ebi-eas-GCST90018588, bbj-a-76, and bbj-a-107. Among them, ebi-eas-GCST90018588 served as the training cohort, and bbj-a-76 and bbj-a-107 were used as the testing cohort.

Bbj-a-76 contains 7,492,477 SNPs and includes a sample size of 33,870 individuals, including 6,692 cases and 27,178 controls (Tanikawa et al., 2018). The two cohorts include both male and female participants from Asia.

Bbj-a-107 contains 8,885,369 SNPs and has a sample size of 202,807 individuals, including 7,062 cases and 195,745 controls. The two cohorts include both male and female participants from Asia.

### Screening of instrumental variables

According to the aforementioned selection criteria, SNPs were extracted from the exposures and outcomes variables for the present analysis. Ultimately, 17 oral flora were found to be causally associated with CRC. 70 SNPs obtained from *s Pauljensenia cellulosilytica*, 73 SNPs from *s Lachnoanaerobaculum*, 83 SNPs from *s F0040*, 81 SNPs from *s Haemophilus*, 74 SNPs from *s Capnocytophaga ochracea*, 83 SNPs from *s Streptococcus mitis AZ*, 67 SNPs from *g Fusobacterium*, 76 SNPs from *s Aggregatibacter*, 82 SNPs from *s Streptococcus sanguinis*, 79 SNPs from *s Streptococcus pneumoniae D,* 77 SNPs from *s TM7x*, 72 SNPs from *s Streptococcus parasanguinis C*, 70 SNPs from *s Campylobacter A concisus F*, 69 SNPs from *s Metamycoplasma salivarium*, 93 SNPs from *s Streptococcus anginosus*, 79 SNPs from *s Mogibacterium pumilum*, and 82 SNPs from *s Neisseria mucosa* were finally included in the MR analysis. The *F* values of these instrumental variables all exceeded 10, indicating the absence of weak instrumental variables. Detailed information on IVs is provided in Supplementary Table S1.

### Two-sample Mendelian randomization analysis

**Figure 1:**
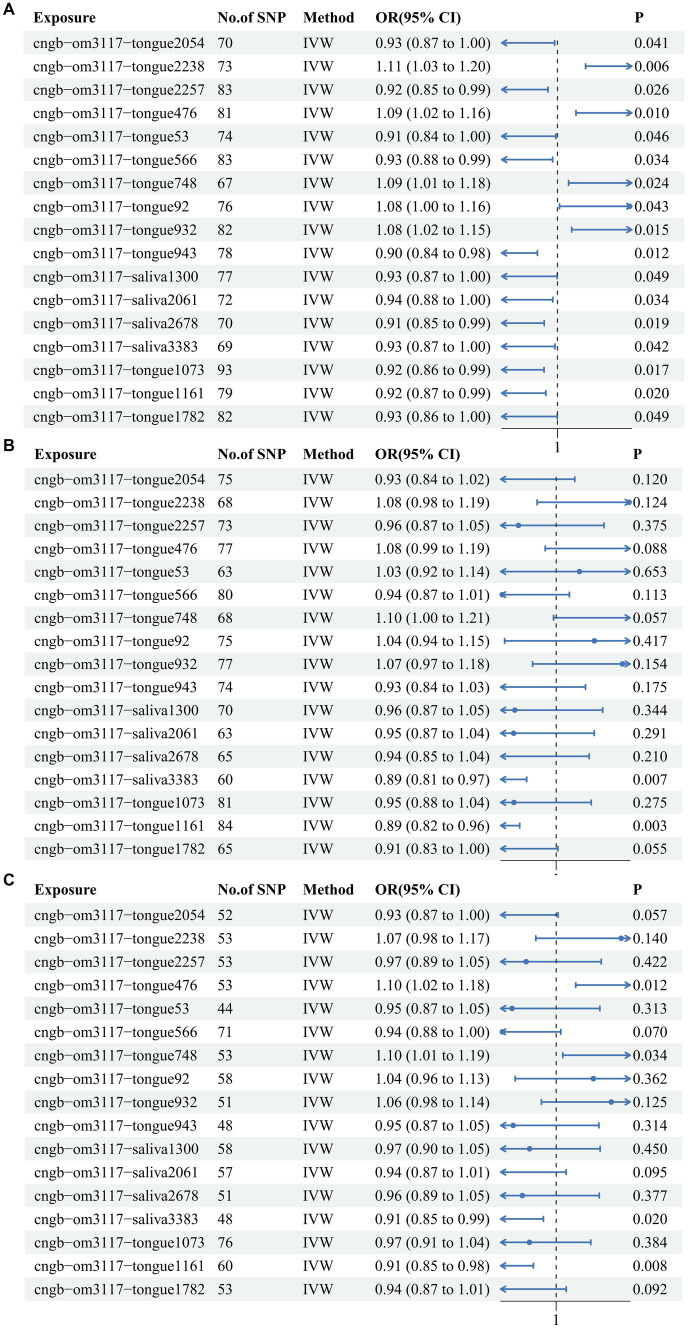
The IVW results for the 17 oral flora associated with CRC in the training cohort and testing cohort. **(A)** ebi-eas-GCST90018588; **(B)** bbj-a-76; **(C)** bbj-a-107.

The IVW results indicated that a total of 17 oral flora were causally associated with CRC. Specifically, *s Pauljensenia cellulosilytica* (IVW OR: 0.929, 95%CI: 0.865–0.997, *p* value: 0.041), *s F0040* (IVW OR: 0.919, 95%CI: 0.852–0.99, *p* value: 0.026), *s Capnocytophaga ochracea* (IVW OR: 0.913, 95%CI: 0.836–0.998, *p* value: 0.046), *s Streptococcus mitis AZ* (IVW OR: 0.934, 95%CI: 0.877–0.995, *p* value: 0.034), *s Streptococcus pneumoniae* D (IVW OR: 0.896, 95%CI: 0.825–0.973, *p* value: 0.009), *s TM7x* (IVW OR: 0.934, 95%CI: 0.873–1, *p* value: 0.049), s *Streptococcus parasanguinis* C (IVW OR: 0.935, 95%CI: 0.879–0.995, *p* value: 0.034), s *Campylobacter A concisus F* (IVW OR: 0.915, 95%CI: 0.849–0.986, *p* value: 0.019), s *Metamycoplasma salivarium* (IVW OR: 0.929, 95%CI: 0.866–0.997, *p* value: 0.042), s *Streptococcus anginosus* (IVW OR: 0.922, 95%CI: 0.862–0.986, *p* value: 0.017), s *Mogibacterium pumilum* (IVW OR: 0.924, 95%CI: 0.865–0.987, *p* value: 0.02), and s *Neisseria mucosa* (IVW OR: 0.929, 95%CI: 0.863–1, *p* value: 0.049) were identified as protective factors for CRC. Conversely, *s Lachnoanaerobaculum* (IVW OR: 1.111, 95%CI: 1.03–1.198, *p* value: 0.006), *s Haemophilus* (IVW OR: 1.087, 95%CI: 1.02–1.159, *p* value: 0.01), *g Fusobacterium* (IVW OR: 1.095, 95%CI: 1.012–1.185, *p* value: 0.024), *s Aggregatibacter* (IVW OR: 1.078, 95%CI: 1.002–1.159, *p* value: 0.043) and *s Streptococcus sanguinis* (IVW OR: 1.083, 95%CI: 1.015–1.155, *p* value: 0.015) were identified as risk factors for CRC (Figure 1A; Supplementary Table S2). This outcome was also evident in the scatter plot generated from the MR analysis, which illustrates the influence of each SNP on the 17 oral flora and CRC and displays the impact of exposure on the outcome (Supplementary Figure S1). Moreover, the Forest plot presents the MR effect size of the 17 oral flora on CRC for each IV. MR Egger and IVW methods were used to calculate the MR effect sizes of all IVs, displayed in red intervals (Supplementary Figure S2). Meanwhile, the heterogeneity test suggested that the included IVs were homogeneous, with a symmetrical distribution on the funnel plot (Supplementary Figure S3; Supplementary Table S3). The leave-one-out sensitivity analysis demonstrated the robustness of the results when each independent variable was systematically excluded (Supplementary Figure S4). Lastly, no evidence of a reverse causal relationship was observed between the 17 oral flora on CRC.

### Verification based on testing cohort

In order to verify the reliability of the training cohort results, TSMR analysis was performed using identical parameters in the testing cohorts. Among them, *s Haemophilus*, *g Fusobacterium*, s *Metamycoplasma salivarium*, and s *Mogibacterium pumilum* were confirmed in 2 testing cohorts. In one of the testing cohorts, *s Metamycoplasma salivarium* (IVW OR: 0.887, 95%CI: 0.812–0.968, *p* value: 0.007) and *s Mogibacterium pumilum* (IVW OR: 0.886, 95%CI: 0.817–0.961, *p* value: 0.003) exerted protective effects against CRC (Figure 1B). In another testing cohort, *Metamycoplasma salivarium* (IVW OR: 0.915, 95%CI: 0.848–0.986, *p* value: 0.02) and *s Mogibacterium pumilum* (IVW OR: 0.91, 95%CI: 0.848–0.975, *p* value: 0.008) exerted protective effects against CRC, whereas *s Haemophilus* (IVW OR: 1.1, 95%CI: 1.021–1.185, *p* value: 0.012) and *g Fusobacterium* (IVW OR: 1.096, 95%CI: 1.007–1.194, *p* value: 0.034) were identified as risk factors for CRC (Figure 1C). All of the results of IVW were showed in heat map (Supplementary Figure S5).

### Meta-analysis based on the inverse-variance weighted method for the training and testing cohorts

**Figure 2:**
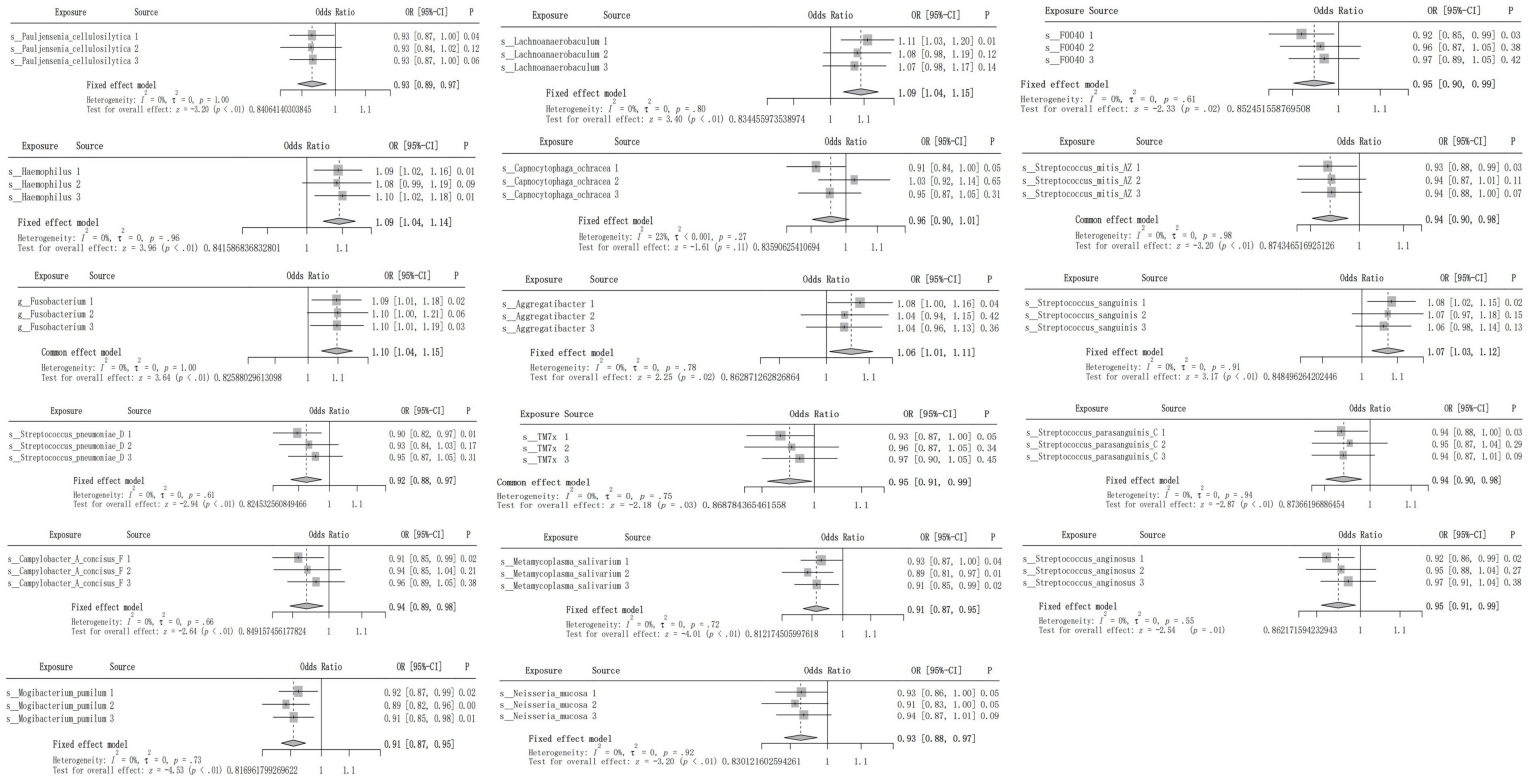
Meta-analysis integrating the IVW results of the 17 oral flora associated with CRC in the training and testing cohorts.

Although causal associations were only noted between CRC and 4 oral flora in the testing cohorts, the meta-analysis demonstrated that 16 oral flora showed a potential causal relationship with CRC, and the differences were statistically significant. Source 1 refers to ebi-eas-GCST90018588, Source 2 refers to bbj-a-76, and Source 3 refers to bbj-a-107 (Figure 2).

### Multivariate Mendelian randomization analysis

**Figure 3:**
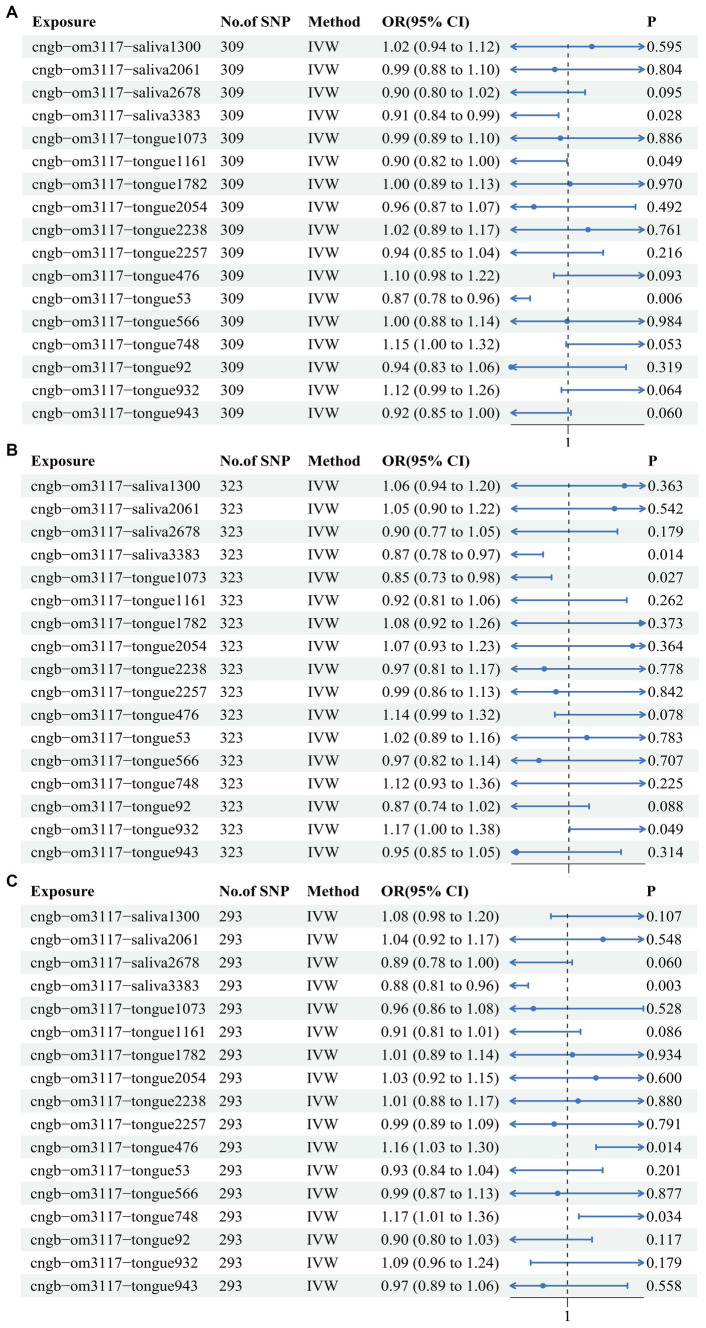
Multivariate Mendelian randomization analysis of the 17 oral flora linked to CRC in the training and testing cohorts. **(A)** ebi-eas-GCST90018588; **(B)** bbj-a-76; **(C)** bbj-a-107.

In order to further elucidate the role of the 17 oral flora in CRC and consolidate the reliability of TSMR and meta-analysis results, MVMR was used to analyze oral flora with positive results. A total of 309 shared IVs were extracted among the 17 oral flora. The results of TSMR for 3 oral flora remained consistent in the MVMR analysis in the training cohort. Among them, *s Metamycoplasma salivarium* (IVW OR: 0.913, 95% CI: 0.842–0.99, *p* value: 0.028), *s Mogibacterium pumilum* (IVW OR: 0.903, 95% CI: 0.816–0.999, *p* value: 0.049), and *s Capnocytophaga ochracea* (IVW OR: 0.867, 95% CI: 0.782–0.96, *p* value: 0.006) were identified as protective factors for CRC (Figure 3A). At the same time, the results of TSMR for 3 oral flora remained stable in the MVMR analysis in one of the testing cohorts. Among them, *s Metamycoplasma salivarium* (IVW OR: 0.873, 95% CI: 0.784–0.973, *p* value: 0.014) and *s Streptococcus anginosus* (IVW OR: 0.849, 95% CI: 0.734–0.982, *p* value: 0.027) was identified as protective factors against CRC, whereas *s Streptococcus sanguinis* (IVW OR: 1.174, 95% CI: 1.001–1.377, *p* value: 0.049) was identified as a risk factor for CRC (Figure 3B). Likewise, the results of TSMR for 3 oral flora remained stable in the MVMR analysis in another testing cohort. Among them, *s Metamycoplasma salivarium* (IVW OR: 0.88, 95% CI: 0.808–0.959, *p* value: 0.003) was identified as a protective factor against CRC, *s Haemophilus* (IVW OR: 1.158, 95% CI: 1.03–1.302, *p* value: 0.014), whereas *g Fusobacterium* (IVW OR: 1.175, 95% CI: 1.012–1.364, *p* value: 0.034) was identified as a risk factor for CRC (Figure 3C). MR Egger and IVW confirmed the absence of heterogeneity in IVs. The Egger intercept was very close to 0, and the *p* value was >0.05, indicating the absence of horizontal pleiotropy (Supplementary Table S4). These results were consistent with those of the MR Presso test (Supplementary Table S5).

## Discussion

Although oral flora has been described to be potentially linked to CRC, this relationship does not appear to be stable, with inconsistencies reported across studies. This study explored the causal association between oral flora and CRC through TSMR analysis and unveiled that 17 oral flora had a potential causal association with CRC in the training cohort. In one of the testing cohorts, a causal relationship was identified between 2 oral flora and CRC. In another testing cohort, a causal relationship was identified between 4 oral flora and CRC. It was worthwhile emphasizing that pooling the results through a meta-analysis yielded 16 oral flora that have a potential causal relationship with CRC. Further MVMR analysis in both training cohort and testing cohorts uncovered that *s Haemophilus*, *g Fusobacterium*, *s Metamycoplasma salivarium*, and *s Mogibacterium pumilum* remained causally associated with CRC. Despite *s Metamycoplasma salivarium* and *s Mogibacterium pumilum* exhibiting causal relationships with CRC, their associations have not been reported in the literature, highlighting them as areas with research potential.

Of note, microbial flora has been reported to be closely related to the occurrence and development of CRC, suggesting its potential as a preventive and therapeutic strategy. In clinical specimens, the presence of specific bacteria such as *Fusobacterium nucleatum*, *Escherichia coliwas*, and *Bacteroides fragilis* were found to be positively associated with increased chemokine expression. However, antibiotic treatment significantly decreased the abundance of these bacteria (Cremonesi et al., 2018). Intestinal microbiota dysbiosis can lead to alterations in T cell phenotypes, resulting in an inflammatory, immunostimulatory, or immunosuppressive phenotype influenced by the tumor microenvironment and microbiota composition (Hou et al., 2022). This interplay may impact the effectiveness of immunotherapy for CRC (Wang et al., 2023). While research on the role of the microbiome and CRC is expanding, the majority of studies have focused on intestinal and fecal flora, leaving a gap in research on oral flora. The oral cavity, as the initial organ of the human digestive system, hosts a diverse population of microorganisms in saliva and on the tongue coating. These microorganisms subsequently migrate to the gastrointestinal tract through ingestion and digestion, playing a decisive role in the pathogenesis of CRC.

Oral pathogens, such as *Fusobacterium*, have been identified as pathogenic agents associated with delayed colonization of oral biofilms and various human diseases, including CRC and juvenile periodontitis. The *Fusobacterium* genus consists of anaerobic gram-negative non-spore-forming bacteria that are frequently present in the oral and intestinal microbiota of humans. This genus exhibits significant diversity, with certain members, particularly *F. nucleatum*, being more abundant in CRC samples and correlated with various pathological conditions (Nosho et al., 2016). Zhang et al. (2022) employed high-throughput sequencing of the 16S rRNA gene V4 region to examine and compare the oral, fecal, and tissue microbiota of 53 individuals with colorectal cancer (CRC) and 70 healthy individuals. Their findings exposed a significant increase in the abundance of *Fusobacterium* in CRC patients compared to healthy controls, as well as the presence of similar and diverse bacterial networks in the oral and tissue microbiota. In another study, Flemer et al. (2018) utilized oropharyngeal swab samples to identify the oral microbiota of CRC patients using comparable detection techniques and concluded that Haemophilus (14.2%) and *Fusobacterium* (5.4%) were the dominant bacterial species in the cohort (Flemer et al., 2018). The potential involvement of *F. nucleatum* in the development and progression of tumors may be attributed to its ability to promote cell proliferation and inhibit immune responses. The presence of these bacteria in tumor tissue has been shown to be positively associated with the increased synthesis and secretion of proinflammatory cytokines such as IL-6, IL-17, and TNF-α (Bostanghadiri et al., 2023), a phenomenon consistent with the activation of nuclear factor kappa B (NF-κB) (Kostic et al., 2013). Furthermore, *F. nucleatum* facilitates the development of colon cancer by releasing bioactive molecules such as short peptides and short-chain fatty acids, which attract myeloid-derived suppressor cells and suppress the activity of CD4+ T-cells. Ultimately, the evasion of tumor cell lysis by NK cells can be achieved through the expression of the Fap2 protein, which interacts with the T cell immunoglobulin and ITIM (TIGIT domain) receptor on NK cells, thereby inhibiting their cytotoxic activity (Bashir et al., 2016).

Furthermore, our research identified *s Haemophilus* as another significant risk factor for CRC, with *Haemophilus influenzae* being the predominant subtype. *H. influenzae* is an oxidase-positive, facultatively anaerobic, non-motile Gram-negative bacillus that commonly inhabits the human respiratory tract and is associated with respiratory illnesses (St Geme, 2002; Murphy et al., 2009; Howard et al., 1988). Among identifiable strains, *H. influenzae* serotype b (Hib) exhibits the highest level of virulence. Huo et al. (2022) investigated the intestinal mucosal microorganisms of CRC patients undergoing surgery and identified a correlation between elevated levels of *Haemophilus* and lower disease-free survival (DFS) or overall survival (OS). According to another study, the prevalence of *Haemophilus* in fecal specimens from individuals with CRC was significantly higher compared to those in the control group (Park et al., 2020). Notably, following surgical excision of the tumor, the prevalence of *Haemophilus* declined, indicating its potential role as an indirect carcinogene (Park et al., 2020). The potential pathogenic impact of *Haemophilus* on CRC may be intricately linked to inflammatory stimulation. A study pointed out that exposure to *Haemophilus* led to pronounced lung inflammation in mice, resulting in a significant elevation in the number of mononuclear cells and neutrophils in the exposed group compared to the control group (Halappanavar et al., 2013). Another study evinced that *Haemophilus* was closely related to the increase in the number of neutrophils in host infections (Wang et al., 2019). Hughes et al. determined that *Haemophilus influenzae* infection can upregulate the expression of macrophage ubiquitin ligase Pellino-1 and induce an inflammatory response through the TLR4 signaling pathway (Hughes et al., 2019).

Taken together, established methods such as MR analysis were employed to identify IVs and ensure their reliability. Furthermore, the results were validated using testing cohort and meta-analysis to achieve relatively objective results. Nevertheless, the present study also has some limitations that cannot be overlooked. Given that all samples were derived from Asian populations, the findings may not be generalizable to other populations. On the other hand, there may be significant variability within this population due to differences in gender, age, and other factors. Thus, further stratification is warranted to evaluate the robustness of our results.

## Conclusion

In this study, 17 oral flora that were causally associated with CRC were identified. Among them, *s Haemophilus*, *g Fusobacterium*, *s Metamycoplasma salivarium*, and *s Mogibacterium pumilum* had the strongest associations, considering that they were validated in the testing cohorts and had significant differences in the MVMR analysis. Further confirmation of the relationships between these 4 oral flora and CRC is required through large-scale randomized controlled trials.

## Data Availability

The original contributions presented in the study are included in the article/Supplementary material, further inquiries can be directed to the corresponding author/s.

## Supplementary material

Supplementary Figure S1 Scatter plot displaying the effect distribution of all SNPs in the training cohort.

Supplementary Figure S2 Forest plot depicting the MR effect sizes of 17 oral flora related to CRC in the training cohort.

Supplementary Figure S3 Funnel plot presenting the distribution of all SNPs in the training cohort.

Supplementary Figure S4 Leave-one-out sensitivity analysis showing that no single SNP significantly impacts the outcome in the training cohort.

Supplementary Figure S5 Heat map of all of the results of IVW method in three cohorts.

Supplementary Table S1 Detailed characteristics of SNPs in the training cohort.

Supplementary Table S2 All MR results for the 17 oral flora associated with CRC in the training cohort.

Supplementary Table S3 Heterogeneity test for the 17 oral flora associated with CRC in the training cohort.

Supplementary Table S4 Pleiotropy test for the 17 oral flora associated with CRC using MR Egger in the training cohort.

Supplementary Table S5 Pleiotropy test for the 17 oral flora associated with CRC using MR Presso in the training cohort.
