# Supplementary figures and images for: Unveiling the hidden link between oral flora and colorectal cancer: a bidirectional Mendelian randomization analysis and meta-analysis
Source: Front Microbiol. 2024 Sep 10;15:1451160. doi: 10.3389/fmicb.2024.1451160 (PMC11420047; doi:10.3389/fmicb.2024.1451160)

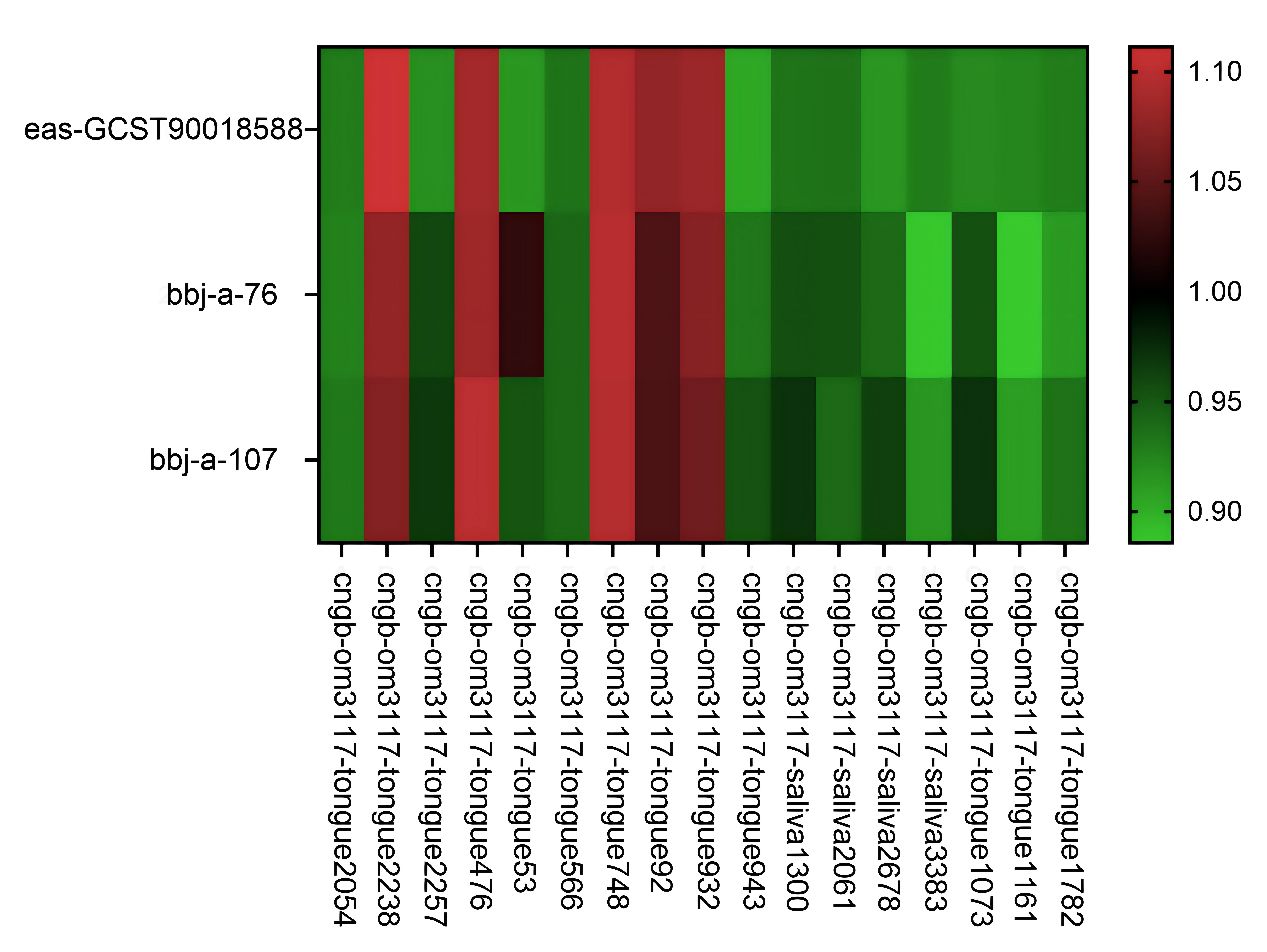

Supplement: Supplementary Figure S5 — Heat map of all of the results of IVW method in three cohorts. [file Image_5.JPEG]
