# Supplementary material for: Unveiling the hidden link between oral flora and colorectal cancer: a bidirectional Mendelian randomization analysis and meta-analysis
Source: Front Microbiol. 2024 Sep 10;15:1451160. doi: 10.3389/fmicb.2024.1451160 (PMC11420047; doi:10.3389/fmicb.2024.1451160)
Supplement: Supplementary Table S3 — Heterogeneity test for the 17 oral flora associated with CRC in the training cohort. [file Table_3.DOCX]

**STROBE-MR checklist of recommended items to address in reports of Mendelian randomization studies**^1^ ^2^

| **Item No.** | **Section** | **Checklist item** | **Page No.** | **Relevant text from manuscript** |
| --- | --- | --- | --- | --- |
| 1 | **TITLE and ABSTRACT** | Indicate Mendelian randomization (MR) as the study’s design in the title and/or the abstract if that is a main purpose of the study | 1-2 | Unveiling the Hidden Link Between Oral Flora and Colorectal Cancer: A Bidirectional Mendelian randomization analysis and meta-analysis |
|  | **INTRODUCTION** |  |  |  |
| 2 | **Background** | Explain the scientific background and rationale for the reported study. What is the exposure? Is a potential causal relationship between exposure and outcome plausible? Justify why MR is a helpful method to address the study question | 72-86 | Oral flora is one of the most complex microenvironments in the human body, containing various microorganisms such as various bacteria and fungi(7). The human oral flora is mainly distributed in the tongue and saliva(8)，and the microorganisms it inhabits can colonize the intestines of susceptible individuals(9,10,11). In fact, a healthy individual swallows 1 - 1.5L of saliva every day, which reaches the human gastrointestinal tract with swallowing(12). Therefore, oral flora is closely related to human health, especially for digestive tract diseases(13). Different studies have shown that the oral cavity is an extragastric reservoir for H. pylori(14,15,16). This may explain the infectious process of H. pylori, which may first colonize the oral cavity and then move further to the stomach(17). Helicobacter pylori infection in the stomach has been considered as one of the high risk factors for gastric cancer(17). For the digestive system, the colorectum usually means the final route of food. After going through the long process from the mouth to the colorectum, the study of changes in oral flora is crucial because it may have an important impact on certain intestinal diseases. |
| 3 | **Objectives** | State specific objectives clearly, including pre-specified causal hypotheses (if any). State that MR is a method that, under specific assumptions, intends to estimate causal effects | 104-110 | Mendelian Randomization (MR) analysis is a method for evaluating causal associations and is widely used in research fields such as epidemiology(23). Because alleles of parental genetic variants are randomly assigned, MR analysis can simulate the assignment method in randomized controlled trials, which can greatly reduce the influence of confounding factors and reverse causality in observational studies, thereby enhancing inferences of causal associations between exposures and outcomes(24). |
|  | **METHODS** |  |  |  |
| 4 | **Study design and data sources** | Present key elements of the study design early in the article. Consider including a table listing sources of data for all phases of the study. For each data source contributing to the analysis, describe the following: | 121-133 | The SNP data of oral flora and CRC were obtained from Asia population. The GWAS summary data of oral flora was derived from the data published by Liu et al. in 2021(25). In this study, the authors conducted a large-scale metagenomic-genome-wide association study (mgGWAS) on 2017 tongue dorsum samples and 1915 saliva samples from 2984 healthy samples, and provided high-depth whole-genome sequencing data. This study included a total of 1549 saliva data and 1568 tongue data. And this study further verified the identified associations in an independently replicated cohort of 1494 individuals.  The SNP data of CRC in training cohort is from BioBank Japan, and these samples were also from Asia(26). In this study, 167,691 samples were included, including 159,386 control samples and 8305 disease samples. The samples included both males and females. Finally, a total of 12,456,388 SNPs were obtained from these samples. |
|  | a) | Setting: Describe the study design and the underlying population, if possible. Describe the setting, locations, and relevant dates, including periods of recruitment, exposure, follow-up, and data collection, when available. | Na |  |
|  | b) | Participants: Give the eligibility criteria, and the sources and methods of selection of participants. Report the sample size, and whether any power or sample size calculations were carried out prior to the main analysis | Na |  |
|  | c) | Describe measurement, quality control and selection of genetic variants | 135-153 | Initially, to acquire instrumental variables (IVs) with strong correlation to oral flora, a filtering threshold of P1=5e-5 was established for single nucleotide polymorphisms (SNPs). SNPs exhibiting greater significance levels are deemed to be closely associated with the heritability of oral flora. Furthermore, an F test was conducted on each IVs to eliminate weak IVs. The F test formula is expressed as F = (Beta/Se)^2, where Beta signifies the effect size of IVs on oral flora, and Se denotes the standard error associated with Beta. IVs with a value of less than 10 in the F test were excluded from the study. Additionally, a linkage disequilibrium assessment was conducted on the instrumental variables. Linkage disequilibrium in genetics denotes the likelihood of alleles from multiple gene loci co-occurring on a single chromosome at a frequency greater than that expected by chance. Such occurrences are incompatible with Mendelian randomization analysis. To mitigate this bias, a threshold of r2 = 0.001 and Kb = 10000 was established.  The IVs of CRC are extracted concurrently utilizing the IVs of the oral flora following a screening process. A threshold of P2=5e-5 is set for the IVs of the outcome in order to eliminate highly correlated variables. Proxy tools are not established for IVs that are not present in the CRC dataset to ensure the accuracy and reliability of the results. Subsequently, the data of IVs from the oral flora and CRC are combined, and palindromic SNPs are eliminated. |
|  | d) | For each exposure, outcome, and other relevant variables, describe methods of assessment and diagnostic criteria for diseases | 135-153 | Initially, to acquire instrumental variables (IVs) with strong correlation to oral flora, a filtering threshold of P1=5e-5 was established for single nucleotide polymorphisms (SNPs). SNPs exhibiting greater significance levels are deemed to be closely associated with the heritability of oral flora. Furthermore, an F test was conducted on each IVs to eliminate weak IVs. The F test formula is expressed as F = (Beta/Se)^2, where Beta signifies the effect size of IVs on oral flora, and Se denotes the standard error associated with Beta. IVs with a value of less than 10 in the F test were excluded from the study. Additionally, a linkage disequilibrium assessment was conducted on the instrumental variables. Linkage disequilibrium in genetics denotes the likelihood of alleles from multiple gene loci co-occurring on a single chromosome at a frequency greater than that expected by chance. Such occurrences are incompatible with Mendelian randomization analysis. To mitigate this bias, a threshold of r2 = 0.001 and Kb = 10000 was established.  The IVs of CRC are extracted concurrently utilizing the IVs of the oral flora following a screening process. A threshold of P2=5e-5 is set for the IVs of the outcome in order to eliminate highly correlated variables. Proxy tools are not established for IVs that are not present in the CRC dataset to ensure the accuracy and reliability of the results. Subsequently, the data of IVs from the oral flora and CRC are combined, and palindromic SNPs are eliminated. |
|  | e) | Provide details of ethics committee approval and participant informed consent, if relevant | Na |  |
| 5 | **Assumptions** | Explicitly state the three core IV assumptions for the main analysis (relevance, independence and exclusion restriction) as well assumptions for any additional or sensitivity analysis | 187-201 | Horizontal pleiotropy significantly impacts the validity of MR analysis findings by introducing the possibility of IVs exerting effects on outcomes through multiple genetic pathways, thereby contravening fundamental MR analysis principles. To identify and address horizontal pleiotropy, we employed Presso (30)and MR Egger methodologies to assess IVs for potential pleiotropic effects. IVs that passed both tests concurrently were deemed free from horizontal pleiotropy, while those exhibiting pleiotropic effects were excluded from the analysis. Furthermore, heterogeneity testing was conducted on IVs using the IVW and MR Egger methods. IVs exhibiting heterogeneity were subsequently excluded from the analysis.  A leave-one-out sensitivity analysis was conducted for each IVs to assess the potential influence of individual SNPs on the outcome. The Steiger test was utilized to identify and exclude SNPs exhibiting reverse causality, as these can distort the interpretation of the relationship between exposure and outcome factors. Additionally, a Reverse Mendelian randomization (RMR) analysis was performed between CRC and oral flora to further validate the findings. |
| 6 | **Statistical methods: main analysis** | Describe statistical methods and statistics used |  |  |
|  | a) | Describe how quantitative variables were handled in the analyses (i.e., scale, units, model) | Na |  |
|  | b) | Describe how genetic variants were handled in the analyses and, if applicable, how their weights were selected | 135-153 | Initially, to acquire instrumental variables (IVs) with strong correlation to oral flora, a filtering threshold of P1=5e-5 was established for single nucleotide polymorphisms (SNPs). SNPs exhibiting greater significance levels are deemed to be closely associated with the heritability of oral flora. Furthermore, an F test was conducted on each IVs to eliminate weak IVs. The F test formula is expressed as F = (Beta/Se)^2, where Beta signifies the effect size of IVs on oral flora, and Se denotes the standard error associated with Beta. IVs with a value of less than 10 in the F test were excluded from the study. Additionally, a linkage disequilibrium assessment was conducted on the instrumental variables. Linkage disequilibrium in genetics denotes the likelihood of alleles from multiple gene loci co-occurring on a single chromosome at a frequency greater than that expected by chance. Such occurrences are incompatible with Mendelian randomization analysis. To mitigate this bias, a threshold of r2 = 0.001 and Kb = 10000 was established.  The IVs of CRC are extracted concurrently utilizing the IVs of the oral flora following a screening process. A threshold of P2=5e-5 is set for the IVs of the outcome in order to eliminate highly correlated variables. Proxy tools are not established for IVs that are not present in the CRC dataset to ensure the accuracy and reliability of the results. Subsequently, the data of IVs from the oral flora and CRC are combined, and palindromic SNPs are eliminated. |
|  | c) | Describe the MR estimator (e.g. two-stage least squares, Wald ratio) and related statistics. Detail the included covariates and, in case of two-sample MR, whether the same covariate set was used for adjustment in the two samples | 155-164 | The TwosampleMR package in the R language was utilized for conducting the comprehensive MR analysis on the merged data. The MR analysis encompassed four distinct methods, including the Inverse Variance Weighted (IVW), MR Egger, Weighted Median, and Weighted Mode. The results obtained from the IVW method were primarily relied upon for evaluation. Notably, the IVW method is advantageous in detecting bias even in the presence of invalid IVs(27). In contrast, the MR Egger method introduces an intercept to assess and address horizontal pleiotropy in IVs(28). For the sake of robustness of the results, we only retain the results with the same Beta direction in the this four analysis methods. It is worth noting that unclassified oral flora was not retained in this study. |
|  | d) | Explain how missing data were addressed | Na |  |
|  | e) | If applicable, indicate how multiple testing was addressed | Na |  |
| 7 | **Assessment of assumptions** | Describe any methods or prior knowledge used to assess the assumptions or justify their validity | 155-164 | The TwosampleMR package in the R language was utilized for conducting the comprehensive MR analysis on the merged data. The MR analysis encompassed four distinct methods, including the Inverse Variance Weighted (IVW), MR Egger, Weighted Median, and Weighted Mode. The results obtained from the IVW method were primarily relied upon for evaluation. Notably, the IVW method is advantageous in detecting bias even in the presence of invalid IVs(27). In contrast, the MR Egger method introduces an intercept to assess and address horizontal pleiotropy in IVs(28). For the sake of robustness of the results, we only retain the results with the same Beta direction in the this four analysis methods. It is worth noting that unclassified oral flora was not retained in this study. |
| 8 | **Sensitivity analyses and additional analyses** | Describe any sensitivity analyses or additional analyses performed (e.g. comparison of effect estimates from different approaches, independent replication, bias analytic techniques, validation of instruments, simulations) | 187-201 | Horizontal pleiotropy significantly impacts the validity of MR analysis findings by introducing the possibility of IVs exerting effects on outcomes through multiple genetic pathways, thereby contravening fundamental MR analysis principles. To identify and address horizontal pleiotropy, we employed Presso (30)and MR Egger methodologies to assess IVs for potential pleiotropic effects. IVs that passed both tests concurrently were deemed free from horizontal pleiotropy, while those exhibiting pleiotropic effects were excluded from the analysis. Furthermore, heterogeneity testing was conducted on IVs using the IVW and MR Egger methods. IVs exhibiting heterogeneity were subsequently excluded from the analysis.  A leave-one-out sensitivity analysis was conducted for each IVs to assess the potential influence of individual SNPs on the outcome. The Steiger test was utilized to identify and exclude SNPs exhibiting reverse causality, as these can distort the interpretation of the relationship between exposure and outcome factors. Additionally, a Reverse Mendelian randomization (RMR) analysis was performed between CRC and oral flora to further validate the findings. |
| 9 | **Software and pre-registration** |  |  |  |
|  | a) | Name statistical software and package(s), including version and settings used | 187-201 | Horizontal pleiotropy significantly impacts the validity of MR analysis findings by introducing the possibility of IVs exerting effects on outcomes through multiple genetic pathways, thereby contravening fundamental MR analysis principles. To identify and address horizontal pleiotropy, we employed Presso (30)and MR Egger methodologies to assess IVs for potential pleiotropic effects. IVs that passed both tests concurrently were deemed free from horizontal pleiotropy, while those exhibiting pleiotropic effects were excluded from the analysis. Furthermore, heterogeneity testing was conducted on IVs using the IVW and MR Egger methods. IVs exhibiting heterogeneity were subsequently excluded from the analysis.  A leave-one-out sensitivity analysis was conducted for each IVs to assess the potential influence of individual SNPs on the outcome. The Steiger test was utilized to identify and exclude SNPs exhibiting reverse causality, as these can distort the interpretation of the relationship between exposure and outcome factors. Additionally, a Reverse Mendelian randomization (RMR) analysis was performed between CRC and oral flora to further validate the findings. |
|  | b) | State whether the study protocol and details were pre-registered (as well as when and where) | Na |  |
|  | **RESULTS** |  |  |  |
| 10 | **Descriptive data** |  |  |  |
|  | a) | Report the numbers of individuals at each stage of included studies and reasons for exclusion. Consider use of a flow diagram | 216-231 | According to the aforementioned selection criteria, single nucleotide polymorphisms (SNPs) were extracted from the exposures and outcomes variables for the present analysis. Ultimately, 17 oral flora were found to be causally associated with CRC. 70 SNPs came from s Pauljensenia cellulosilytica, 73 SNPs came from s Lachnoanaerobaculum, 83 SNPs came from s F0040, 81 SNPs came from s Haemophilus, 74 SNPs came from s Capnocytophaga ochracea, 83 SNPs came from s Streptococcus mitis AZ, 67 SNPs came from g Fusobacterium, 76 SNPs came from s Aggregatibacter, 82 SNPs came from s Streptococcus sanguinis, 79 SNPs came from s Streptococcus pneumoniae D, 77 SNPs came from s TM7x, 72 SNPs came from s Streptococcus parasanguinis C, 70 SNPs came from s Campylobacter A concisus F, 69 SNPs came from s Metamycoplasma salivarium, 93 SNPs came from s Streptococcus anginosus, 79 SNPs came from s Mogibacterium pumilum, and 82 SNPs came from s Neisseria mucosa were finally included in the MR analysis. The F values of these instrumental variables were all >10, indicating that there were no weak instrumental variables. The detailed information of IVs was showed in Supplemental Table 1. |
|  | b) | Report summary statistics for phenotypic exposure(s), outcome(s), and other relevant variables (e.g. means, SDs, proportions) | 233-251 | he IVW results indicated that a total of 17 oral flora are causally associated with CRC. Specially, we found that s Pauljensenia cellulosilytica (IVW OR: 0.929, 95%CI: 0.865-0.997, P value: 0.041), s F0040 (IVW OR: 0.919, 95%CI: 0.852-0.99, P value: 0.026), s Capnocytophaga ochracea (IVW OR: 0.913, 95%CI: 0.836-0.998, P value: 0.046), s Streptococcus mitis AZ (IVW OR: 0.934, 95%CI: 0.877-0.995, P value: 0.034), s Streptococcus pneumoniae D (IVW OR: 0.896, 95%CI: 0.825-0.973, P value: 0.009), s TM7x (IVW OR: 0.934, 95%CI: 0.873-1, P value: 0.049), s Streptococcus parasanguinis C (IVW OR: 0.935, 95%CI: 0.879-0.995, P value: 0.034), s Campylobacter A concisus F (IVW OR: 0.915, 95%CI: 0.849-0.986, P value: 0.019), s Metamycoplasma salivarium (IVW OR: 0.929, 95%CI: 0.866-0.997, P value: 0.042), s Streptococcus anginosus (IVW OR: 0.922, 95%CI: 0.862-0.986, P value: 0.017), s Mogibacterium pumilum (IVW OR: 0.924, 95%CI: 0.865-0.987, P value: 0.02), and s Neisseria mucosa (IVW OR: 0.929, 95%CI: 0.863-1, P value: 0.049) were the protective factors for CRC. While s Lachnoanaerobaculum (IVW OR: 1.111, 95%CI: 1.03-1.198, P value: 0.006), s Haemophilus (IVW OR: 1.087, 95%CI: 1.02-1.159, P value: 0.01), g Fusobacterium (IVW OR: 1.095, 95%CI: 1.012-1.185, P value: 0.024), s Aggregatibacter (IVW OR: 1.078, 95%CI: 1.002-1.159, P value: 0.043) and s Streptococcus sanguinis (IVW OR: 1.083, 95%CI: 1.015-1.155, P value: 0.015) were the risk factors for CRC. Figure1 A. Supplement Table 2. |
|  | c) | If the data sources include meta-analyses of previous studies, provide the assessments of heterogeneity across these studies | 279-283 | Although only 4 oral flora were confirmed casual relationship on CRC in testing cohorts, after integrated the results of IVW method to conduct meta analysis, a total of 16 oral flora showed potential causal relationship on CRC with significant differences. Source 1 refers to ebi-eas-GCST90018588, Source 2 refers to bbj-a-76, and Source 3 refers to bbj-a-107. Figure 2. |
|  | d) | For two-sample MR:  i.  Provide justification of the similarity of the genetic variant-exposure associations between the exposure and outcome samples  ii.  Provide information on the number of individuals who overlap between the exposure and outcome studies | 216-231 | According to the aforementioned selection criteria, single nucleotide polymorphisms (SNPs) were extracted from the exposures and outcomes variables for the present analysis. Ultimately, 17 oral flora were found to be causally associated with CRC. 70 SNPs came from s Pauljensenia cellulosilytica, 73 SNPs came from s Lachnoanaerobaculum, 83 SNPs came from s F0040, 81 SNPs came from s Haemophilus, 74 SNPs came from s Capnocytophaga ochracea, 83 SNPs came from s Streptococcus mitis AZ, 67 SNPs came from g Fusobacterium, 76 SNPs came from s Aggregatibacter, 82 SNPs came from s Streptococcus sanguinis, 79 SNPs came from s Streptococcus pneumoniae D, 77 SNPs came from s TM7x, 72 SNPs came from s Streptococcus parasanguinis C, 70 SNPs came from s Campylobacter A concisus F, 69 SNPs came from s Metamycoplasma salivarium, 93 SNPs came from s Streptococcus anginosus, 79 SNPs came from s Mogibacterium pumilum, and 82 SNPs came from s Neisseria mucosa were finally included in the MR analysis. The F values of these instrumental variables were all >10, indicating that there were no weak instrumental variables. The detailed information of IVs was showed in Supplemental Table 1. |
| 11 | **Main results** |  |  |  |
|  | a) | Report the associations between genetic variant and exposure, and between genetic variant and outcome, preferably on an interpretable scale | 233-251 | The IVW results indicated that a total of 17 oral flora are causally associated with CRC. Specially, we found that s Pauljensenia cellulosilytica (IVW OR: 0.929, 95%CI: 0.865-0.997, P value: 0.041), s F0040 (IVW OR: 0.919, 95%CI: 0.852-0.99, P value: 0.026), s Capnocytophaga ochracea (IVW OR: 0.913, 95%CI: 0.836-0.998, P value: 0.046), s Streptococcus mitis AZ (IVW OR: 0.934, 95%CI: 0.877-0.995, P value: 0.034), s Streptococcus pneumoniae D (IVW OR: 0.896, 95%CI: 0.825-0.973, P value: 0.009), s TM7x (IVW OR: 0.934, 95%CI: 0.873-1, P value: 0.049), s Streptococcus parasanguinis C (IVW OR: 0.935, 95%CI: 0.879-0.995, P value: 0.034), s Campylobacter A concisus F (IVW OR: 0.915, 95%CI: 0.849-0.986, P value: 0.019), s Metamycoplasma salivarium (IVW OR: 0.929, 95%CI: 0.866-0.997, P value: 0.042), s Streptococcus anginosus (IVW OR: 0.922, 95%CI: 0.862-0.986, P value: 0.017), s Mogibacterium pumilum (IVW OR: 0.924, 95%CI: 0.865-0.987, P value: 0.02), and s Neisseria mucosa (IVW OR: 0.929, 95%CI: 0.863-1, P value: 0.049) were the protective factors for CRC. While s Lachnoanaerobaculum (IVW OR: 1.111, 95%CI: 1.03-1.198, P value: 0.006), s Haemophilus (IVW OR: 1.087, 95%CI: 1.02-1.159, P value: 0.01), g Fusobacterium (IVW OR: 1.095, 95%CI: 1.012-1.185, P value: 0.024), s Aggregatibacter (IVW OR: 1.078, 95%CI: 1.002-1.159, P value: 0.043) and s Streptococcus sanguinis (IVW OR: 1.083, 95%CI: 1.015-1.155, P value: 0.015) were the risk factors for CRC. |
|  | b) | Report MR estimates of the relationship between exposure and outcome, and the measures of uncertainty from the MR analysis, on an interpretable scale, such as odds ratio or relative risk per SD difference | Na |  |
|  | c) | If relevant, consider translating estimates of relative risk into absolute risk for a meaningful time period | Na |  |
|  | d) | Consider plots to visualize results (e.g. forest plot, scatterplot of associations between genetic variants and outcome versus between genetic variants and exposure) | 251-254 | Figure1 A. Supplement Table 2. This outcome was also evident in the scatter plot generated from MR analysis. The scatter plot illustrates the influence of each SNP on 17 oral flora and CRC, allowing for a visual representation of the impact of exposure on the outcome. |
| 12 | **Assessment of assumptions** |  |  |  |
|  | a) | Report the assessment of the validity of the assumptions | 257-263 | Heterogeneity testing indicated that the included IVs were homogeneous, and the distribution on the funnel plot was symmetrical. Supplement Figure 3. Supplement Table 3. The leave-one-out sensitivity analysis demonstrated the robustness of the results when each independent variable was systematically eliminated. Supplement Figure 4. Additionally, no evidence of a reverse causal relationship was observed between 17 oral flora on CRC. |
|  | b) | Report any additional statistics (e.g., assessments of heterogeneity across genetic variants, such as *I^2^*, Q statistic or E-value) | 228-231 | The F values of these instrumental variables were all >10, indicating that there were no weak instrumental variables. The detailed information of IVs was showed in Supplemental Table 1. |
| 13 | **Sensitivity analyses and additional analyses** |  |  |  |
|  | a) | Report any sensitivity analyses to assess the robustness of the main results to violations of the assumptions | 257-263 | Heterogeneity testing indicated that the included IVs were homogeneous, and the distribution on the funnel plot was symmetrical. Supplement Figure 3. Supplement Table 3. The leave-one-out sensitivity analysis demonstrated the robustness of the results when each independent variable was systematically eliminated. Supplement Figure 4. Additionally, no evidence of a reverse causal relationship was observed between 17 oral flora on CRC. |
|  | b) | Report results from other sensitivity analyses or additional analyses | 257-263 | Heterogeneity testing indicated that the included IVs were homogeneous, and the distribution on the funnel plot was symmetrical. Supplement Figure 3. Supplement Table 3. The leave-one-out sensitivity analysis demonstrated the robustness of the results when each independent variable was systematically eliminated. Supplement Figure 4. Additionally, no evidence of a reverse causal relationship was observed between 17 oral flora on CRC. |
|  | c) | Report any assessment of direction of causal relationship (e.g., bidirectional MR) | 257-263 | Heterogeneity testing indicated that the included IVs were homogeneous, and the distribution on the funnel plot was symmetrical. Supplement Figure 3. Supplement Table 3. The leave-one-out sensitivity analysis demonstrated the robustness of the results when each independent variable was systematically eliminated. Supplement Figure 4. Additionally, no evidence of a reverse causal relationship was observed between 17 oral flora on CRC. |
|  | d) | When relevant, report and compare with estimates from non-MR analyses | Na |  |
|  | e) | Consider additional plots to visualize results (e.g., leave-one-out analyses) | 257-263 | Heterogeneity testing indicated that the included IVs were homogeneous, and the distribution on the funnel plot was symmetrical. Supplement Figure 3. Supplement Table 3. The leave-one-out sensitivity analysis demonstrated the robustness of the results when each independent variable was systematically eliminated. Supplement Figure 4. Additionally, no evidence of a reverse causal relationship was observed between 17 oral flora on CRC. |
|  | **DISCUSSION** |  |  |  |
| 14 | **Key results** | Summarize key results with reference to study objectives | 308-321 | Although oral flora has been shown to have potential links on CRC, this relationship does not appear to be stable, as in some studies their reported results were inconsistent. In this study, we explored the causal association between oral flora and CRC through TSMR analysis. Our study found that in the training cohort, 17 oral flora had a potential causal association with CRC. In one of the testing cohorts, 2 oral flora showed causal relationship on CRC. In another testing cohort, 4 oral flora showed causal relationship on CRC. It was worth mentioning that after we integrated the results through a meta-analysis, there were still 16 oral flora that have a potential causal relationship with CRC. Further MVMR analysis in both training cohort and testing cohorts showed that s Haemophilus, g Fusobacterium, s Metamycoplasma salivarium and s Mogibacterium pumilum were still causally associated with CRC. Although s Metamycoplasma salivarium and s Mogibacterium pumilum showed causal relationship with CRC, the research on them and CRC has not been reported yet, which is an area with potential. |
| 15 | **Limitations** | Discuss limitations of the study, taking into account the validity of the IV assumptions, other sources of potential bias, and imprecision. Discuss both direction and magnitude of any potential bias and any efforts to address them | 387-391 | All in all, for the screening of IVs, we adopted the current mature methods of MR Analysis to ensure the reliability of IVs. For the conclusion inference, we conducted testing cohort and meta-analysis to verify, in order to obtain a relatively objective result. Of course, this study also has some limitations, all the samples are from Europe, and the conclusions may not be applicable to other populations. |
| 16 | **Interpretation** |  |  |  |
|  | a) | Meaning: Give a cautious overall interpretation of results in the context of their limitations and in comparison with other studies | 308-321 | Although oral flora has been shown to have potential links on CRC, this relationship does not appear to be stable, as in some studies their reported results were inconsistent. In this study, we explored the causal association between oral flora and CRC through TSMR analysis. Our study found that in the training cohort, 17 oral flora had a potential causal association with CRC. In one of the testing cohorts, 2 oral flora showed causal relationship on CRC. In another testing cohort, 4 oral flora showed causal relationship on CRC. It was worth mentioning that after we integrated the results through a meta-analysis, there were still 16 oral flora that have a potential causal relationship with CRC. Further MVMR analysis in both training cohort and testing cohorts showed that s Haemophilus, g Fusobacterium, s Metamycoplasma salivarium and s Mogibacterium pumilum were still causally associated with CRC. Although s Metamycoplasma salivarium and s Mogibacterium pumilum showed causal relationship with CRC, the research on them and CRC has not been reported yet, which is an area with potential. |
|  | b) | Mechanism: Discuss underlying biological mechanisms that could drive a potential causal relationship between the investigated exposure and the outcome, and whether the gene-environment equivalence assumption is reasonable. Use causal language carefully, clarifying that IV estimates may provide causal effects only under certain assumptions | 322-386 | In fact, microbial flora has been reported to be closely related to the occurrence and development of CRC and may become a potential prevention and treatment strategy. In clinical specimens, the presence of specific bacteria such as Fusobacterium nucleatum, Escherichia coliwas and Bacteroides fragilis were found to be positively associated with increased chemokine expression. After antibiotic treatment, the numbers of these flora were significantly reduced(32). Intestinal microbiota dysbiosis can lead to alterations in T cell phenotypes, resulting in an inflammatory, immunostimulatory, or immunosuppressive phenotype, influenced by the tumor microenvironment and composition of the microbiota(33). This interplay may serve as a potential effect in the effectiveness of immunotherapy for CRC(34). While research on the microbiome and CRC is expanding, the majority of studies have concentrated on intestinal and fecal flora, leaving a notable gap in research on oral flora. The oral cavity serves as the initial organ of the human digestive system, housing a significant population of microorganisms in saliva and tongue coating. These microorganisms are subsequently transferred to the gastrointestinal tract through ingestion and digestion, playing a crucial role in the development of CRC.  Oral pathogens, such as Fusobacterium, have been identified as pathogenic agents associated with delayed colonization of oral biofilms and various human diseases, including CRC and juvenile periodontitis. The Fusobacterium genus consists of anaerobic gram-negative non-spore-forming bacteria that are frequently present in the oral and intestinal microbiota of humans. This genus exhibits significant diversity, with certain members, notably F. nucleatum, showing an increased presence in CRC samples and a correlation with various pathological conditions(35). Zhang et al employed high-throughput sequencing of the 16S rRNA gene V4 region to examine and compare the oral, fecal, and tissue microbiota of 53 individuals with colorectal cancer (CRC) and 70 healthy individuals. Their findings revealed a significant elevation of Fusobacterium in CRC patients relative to healthy controls, as well as the presence of similar and diverse bacterial networks in the oral and tissue microbiota(36). In a separate study, Flemer B et al utilized oropharyngeal swab samples to identify the oral microbiota of CRC patients using comparable detection techniques, revealing that Haemophilus (14.2%) and Fusobacterium (5.4%) were two bacterial species that exhibited a higher proportion within the cohort(37). The potential involvement of F. nucleatum in the development and progression of tumors may be attributed to its ability to promote cell proliferation and inhibit immune responses. The presence of these bacteria in tumor tissue has been shown to be positively associated with the increased production of proinflammatory cytokines such as IL-6, IL-17, and TNF-α(38), a phenomenon that aligns with the activation of nuclear factor kappa B (NF-κB)(39). Furthermore, F. nucleatum has been implicated in the promotion of colon cancer by releasing bioactive molecules like short peptides and short-chain fatty acids, which attract myeloid-derived suppressor cells and suppress the activity of CD4+ T-cells. Ultimately, the evasion of tumor cell lysis by NK cells can be achieved through the expression of the Fap2 protein, which interacts with the T cell immunoglobulin and ITIM (TIGIT domain) receptor on NK cells, thereby suppressing their cytotoxic activity(40).  Furthermore, our research has identified s Haemophilus as another significant risk factor for CRC, with Haemophilus influenzae being the predominant subtype. H. influenzae is characterized as an oxidase-positive, facultatively anaerobic, non-motile Gram-negative bacillus that commonly inhabits the human respiratory tract and is associated with respiratory illnesses(41,42,43). Among identifiable strains, H. influenzae serotype b (Hib) exhibits the highest level of virulence. Huo R-X et al. conducted a study on the intestinal mucosal microorganisms of CRC patients undergoing surgery, revealing a correlation between elevated levels of Haemophilus and diminished disease-free survival (DFS) or overall survival (OS)(44). In a separate investigation, the prevalence of Haemophilus in fecal specimens from individuals with CRC was notably elevated compared to those in the control group(45). Notably, following surgical excision of the tumor, the prevalence of Haemophilus declined, indicating its potential role as an indirect carcinogenic agent(45). The potential pathogenic impact of Haemophilus on CRC may be intricately linked to inflammatory stimulation. A study demonstrated that exposure to Haemophilus led to pronounced lung inflammation in mice, resulting in a significant elevation in the number of mononuclear cells and neutrophils in the exposed group compared to the control group(46). In another study, Haemophilus was also closely related to the increase of neutrophils in host infection(47). Hughes et al found that Haemophilus influenzae infection can upregulate the expression level of macrophage ubiquitin ligase Pellino-1 and induce inflammatory response through the TLR4 signaling pathway(48). |
|  | c) | Clinical relevance: Discuss whether the results have clinical or public policy relevance, and to what extent they inform effect sizes of possible interventions | 322-337 | In fact, microbial flora has been reported to be closely related to the occurrence and development of CRC and may become a potential prevention and treatment strategy. In clinical specimens, the presence of specific bacteria such as Fusobacterium nucleatum, Escherichia coliwas and Bacteroides fragilis were found to be positively associated with increased chemokine expression. After antibiotic treatment, the numbers of these flora were significantly reduced(32). Intestinal microbiota dysbiosis can lead to alterations in T cell phenotypes, resulting in an inflammatory, immunostimulatory, or immunosuppressive phenotype, influenced by the tumor microenvironment and composition of the microbiota(33). This interplay may serve as a potential effect in the effectiveness of immunotherapy for CRC(34). While research on the microbiome and CRC is expanding, the majority of studies have concentrated on intestinal and fecal flora, leaving a notable gap in research on oral flora. The oral cavity serves as the initial organ of the human digestive system, housing a significant population of microorganisms in saliva and tongue coating. These microorganisms are subsequently transferred to the gastrointestinal tract through ingestion and digestion, playing a crucial role in the development of CRC. |
| 17 | **Generalizability** | Discuss the generalizability of the study results (a) to other populations, (b) across other exposure periods/timings, and (c) across other levels of exposure | 387-399 | All in all, for the screening of IVs, we adopted the current mature methods of MR Analysis to ensure the reliability of IVs. For the conclusion inference, we conducted testing cohort and meta-analysis to verify, in order to obtain a relatively objective result. Of course, this study also has some limitations, all the samples are from Europe, and the conclusions may not be applicable to other populations.  Conclusion  In this study, we found 17 oral flora that are causally associated with CRC. Among them, s Haemophilus, g Fusobacterium, s Metamycoplasma salivarium and s Mogibacterium pumilum are most closely related to CRC because they have been verified in the testing cohorts and also have significant differences in MVMR. Further confirmation of the relationship between these 4 oral flora and CRC still requires large-scale randomized controlled trials. |
|  | **OTHER INFORMATION** |  |  |  |
| 18 | **Funding** | Describe sources of funding and the role of funders in the present study and, if applicable, sources of funding for the databases and original study or studies on which the present study is based | 410-412 | This study was supported by the National Natural Science Foundation of China (Grant no. 82071413). |
| 19 | **Data and data sharing** | Provide the data used to perform all analyses or report where and how the data can be accessed, and reference these sources in the article. Provide the statistical code needed to reproduce the results in the article, or report whether the code is publicly accessible and if so, where | 403-405 | The data of this study were acquired from GWAS summary data and BioBank Japan database. |
| 20 | **Conflicts of Interest** | All authors should declare all potential conflicts of interest | 407-408 | The authors declared that they have no competing interests. |

This checklist is copyrighted by the Equator Network under the Creative Commons Attribution 3.0 Unported (CC BY 3.0) license.

1. Skrivankova VW, Richmond RC, Woolf BAR, Yarmolinsky J, Davies NM, Swanson SA, et al. Strengthening the Reporting of Observational Studies in Epidemiology using Mendelian Randomization (STROBE-MR) Statement. JAMA. 2021;under review.

2. Skrivankova VW, Richmond RC, Woolf BAR, Davies NM, Swanson SA, VanderWeele TJ, et al. Strengthening the Reporting of Observational Studies in Epidemiology using Mendelian Randomisation (STROBE-MR): Explanation and Elaboration. BMJ. 2021;375:n2233.
